# 非小细胞肺癌脑转移的靶向和免疫治疗

**DOI:** 10.3779/j.issn.1009-3419.2016.08.08

**Published:** 2016-08-20

**Authors:** 琪 宋, 顺昌 焦, 方 李

**Affiliations:** 100853 北京, 解放军总医院肿瘤内一科 Oncology Department, General Hospital of PLA, Beijing 100853, China

**Keywords:** 肺肿瘤, 脑转移, 靶向治疗, 免疫治疗, Lung neoplasms, Brain metastasis, Immunotherapy, Targeted therapy

## Abstract

脑转移是非小细胞肺癌(non-small cell lung cancer, NSCLC)常见并发症, 发生率为30%-50%, 大大影响了患者的生存质量。NSCLC一旦发生脑转移预后极差, 未经治疗者的中位生存期仅为1个月-2个月。基于肺癌驱动基因的靶向治疗为肺癌脑转移提供了新的方法。目前免疫治疗已成为肿瘤治疗的新方向, 它能通过刺激机体免疫系统提高抗肿瘤免疫效应。临床上靶向治疗和免疫治疗的结合运用可使患者获益。

## Abstract (en)

Brain metastasis, a common complication of non-small cell lung cancer (NSCLC) with an incidence rate of 30%-50%, significantly affects the patients' quality of life.The prognosis of patients of NSCLC with brain metastasis is extremely poor, the average median survival is only 1 m-2 m without treatment.The targeted therapy based on lung cancer driven gene is a new treatment.Besides, the immunotherapy which can enhance the effect of anti-cancer by simulating the immune system is a new approach.The combination of targeted therapy and immunotherapy can greatly benefit patients in clinical work.

## 1 病例资料

张某某, 女, 40岁。2009年查体发现左肺占位, 于2009年5月25日行手术治疗, 术后病理:左肺上叶中分化腺癌, 肿瘤大小约2.7 cm×2.6 cm×2 cm, 癌组织侵犯但未侵破肺膜, 未侵犯支气管, 支气管断端未见癌, 肺门淋巴结及送检(第三、五、六、十组)淋巴结均未见转移癌(0/3, 0/1, 0/2, 0/1, 0/2)。免疫组化染色显示肿瘤细胞:HER-1(+++), HER-2(+), p53(+ > 75%), p170(-), Ki-67(+ < 25%), 血管内皮生长因子(+), Top-IIa(+ < 25%), p16(-)。术后行表皮生长因子受体基因检测:19外显子缺失(del E746-A750)、20外显子同义突变(CAG-CAA, Q787Q)、18及21外显子未见突变。术后未行辅助放化疗。2013年5月再次行基因检测:血表皮生长因子受体T790M(-)、组织ALK(-)、雌激素受体beta浆表达(弱+)。其病史及治疗方案见表 1。

**1:** 治疗史 Treatment history

Time of changing treatment regimen	Reason of changing treatment regimen	Treatment regimen and treatment evaluation
2011.10	Liver, lung and brain metastasis	PPy^*^6 cycle (2011.11.28-2012.3.20)
2012.4	Increased liver metastasis and emerging new liver metastasis	Erlotinib 50 mg PO QD (2012.4-2012.10) Erlotinib 600 mg PO QD (2012.10-2012.11)
2012.11	Increased liver metastasis	Nab-Paclitaxely^*^6 cycle (2012.11-2013.2) PR after 2 cycles and SD after 4 cycles
2013.5	Increased liver metastasis and emerging new liver metastasis, a little increased lung metastasis	Afatinib 40 mg PO QD (2013.5-2013.11) Brain MRI (2013.6, 2013.9, 2013.11):brain metastasis CR Liver MRI (2013.9):liver metastasis PD
2013.9	PD in liver and lung metastasis	Interventional treatment for liver and lung with gemcitabine, endostar and nedaplatin
2013.11	Enlarged lung metastasis, a little decreased liver metastasis, a little increased CEA level(10.46 ng/mL-14.43 ng/mL)	Radiotherapy for lung and liver metastasis (2013.12.29-2014.2.4)
2014.4	PD in liver and brain metastasis	Docetaxel+bevacizumaby^*^2 cycles (2014.4.24-2014.5.16)
2014.6	PD in lung, liver and brain metastasis	Afatinib 40 mg PO QD (2014.6-2014.7) Accompanied by side-effects of increased ALT, gastric pain and bone pain
2014.7	Intolerance the side effects of afatinib	Erlotinib+4002 (clinical trial drug) (2014.7-2015.3) Brain MRI (2014-8-29):CR Lung CT and liver MRI:decreased metastasis T10 vertebrae metastasis
2015.1	PD in brain and bone metastasis	Radiotherapy for bone (2015.2-2015.3) WBRT (2015.5-2015.6) Brain MRI (2015-7-28):PR AZD9291 80 mg PO QD (2015.3-2015.9) AZD9291+184 (clinical trial drug, dosage unknown)
2015.9	A little enlargement of lung metastasis	Pause treatment and have a recovery
2015.12	PD in lung metastasis, new lymph nodes metastasis in mediastinum and hilar, new metastasis in 1 lumbar vertebrae	Erlotinib 50 mg PO QD (2015.11.27-2015.12.11) AZD9291+184 (2015.12.11-2015.12.23, dosage unknown)
2015.12	PD in lung metastasis	Apatinib 850 mg QD (2015.12.24-now)
2016.2	PD in lung and liver metastasis	PD-1 antibodyy^*^6 (2016.2-now, 1/2 weeks)
PP:pemetrexed+cisplatin; PR:partial response; SD:stable disease; PD:progressive disease; CR:complete response; WBRT:whole brain radiation therapy.		

## 2 结果

该患者术后分期较早(pT2N0M0 Ib期), 手术为根治性手术, 术后2年余即出现肝、脑、肺等多脏器转移。病情进展后先后给予了化疗、靶向治疗、介入、放疗及免疫治疗, 目前术后生存期已达7年, 可以认为与近年来肺癌治疗技术及新药的发展密不可分。

## 3 讨论

脑转移是非小细胞肺癌(non-small cell lung cancer, NSCLC)常见并发症, 发生率为30%-50%^[1]^, 大大影响了患者的生存质量。NSCLC一旦发生脑转移预后极差, 未经治疗者的中位生存期仅为1个月-2个月^[2]^。纵观该患者脑转移治疗史:术后2年余即出现颅内单发转移, 先后接受了PP方案(pemetrexed+cisplatin)化疗、厄洛替尼靶向治疗、白蛋白紫杉醇化疗及阿法替尼靶向治疗。其中在口服厄洛替尼期间颅内转移灶曾一度略缩小, 口服阿法替尼期间颅内转移灶消失并持续5个月之久。2014年4月脑部病灶进展后, 行2周期多西他赛+贝伐单抗化疗, 疗效评价为疾病进展; 换方案口服厄洛替尼及试验药物(4002), 颅内病灶明显缩小, 疗效评价为部分缓解。2015年1月颅内病灶再次进展后行全脑放疗, 肿瘤缩小, 疗效评价为部分缓解。2016年2月起使用程序性死亡受体1(programmed death 1, PD-1)单抗, 未行影像学检查。

该患者脑转移灶治疗特点为对靶向药物敏感, 曾先后使用厄洛替尼、阿法替尼及试验药物4002, 均取得较好的疗效。与回顾性临床研究吉非替尼和厄洛替尼治疗NSCLC脑转移有一定疗效^[3, 4]^的结论一致。该疗效可能与表皮生长因子受体酪氨酸激酶抑制剂(epidermal growth factor receptor-tyrosine kinase inhibitor, EGFR-TKI)可通过血脑屏障相关, 亦有研究^[5]^表明EGFR-TKI作为小分子化合物可部分通过血脑屏障, 且其在脑转移患者脑脊液中的通透率高于无脑转移患者。

全脑放疗是NSCLC脑转移治疗的标准方案, 包括全脑放射治疗(whole-brain radiation therapy, WBRT)、立体定向放射外科(stereotactic radio surgery, SRS)等, 或者与手术治疗相结合。已有多项系统分析证明WBRT联合靶向药物治疗晚期NSCLC伴多发脑转移的疗效好。但该患者单独使用靶向药物和WBRT时亦取得了较好的疗效。

在NSCLC脑转移患者中, 化疗很少作为一线治疗方案使用, 尤其作为单一治疗模式。目前比较公认的原因之一是由于血脑屏障(blood brain barrier, BBB)的存在, 药物进入颅内有限。针对该患者的脑转移病灶, 化疗药物均未取得明显疗效。

值得关注的是患者在长期综合治疗后使用了目前最新的免疫治疗PD-1单抗。PD-1是一种重要的免疫抑制分子, 是调节免疫细胞功能的一个关键哨所。目前已上市的针对PD-1的抗体为Opdivo和Keytruda。免疫治疗对脑转移的疗效并不十分清楚, 因为大部分临床试验将活动性脑损伤纳为排除标准。一项Keytruda治疗黑色素瘤脑转移的Ⅱ期临观试验(NCT02085070)曾报道Keytruda^[6]^对于脑转移病灶有效, 且起初时MRI表现为一过性的假进展, 后经病理证实转移灶内有少量肿瘤细胞簇, 且伴有出血、反应性星状细胞、炎症细胞及小胶质细胞。今年*Lancet Oncol*发表了一项Keytruda治疗初治的黑色素瘤脑转移和NSCLC脑转移患者的早期数据^[7]^, 其有效率分别为22%和33%, 且免疫治疗疗效持久。提示系统性免疫治疗对于初治或脑转移进展期患者亦有一定疗效。本例患者使用PD-1单抗后自觉体力恢复, 但并未行脑部影响学检查, 我们将会继续进行密切随访。

在临床中大部分脑转移患者最终死于全身疾病的进展, 而不是颅内病灶未控制。因此NSCLC脑转移患者颅内转移灶的治疗与全身治疗是协调统一的, 相信随着靶向、免疫治疗的面纱逐渐被揭开, 将会有更多NSCLC脑转移患者从中获益。
